# Comparing alcohol with tobacco indicates that it is time to move beyond tobacco exceptionalism

**DOI:** 10.1093/eurpub/cky227

**Published:** 2019-03-20

**Authors:** Jim McCambridge, Stephanie Morris

**Affiliations:** Department of Health Sciences, ARRC Building, University of York, Heslington, York, UK

Tobacco is distinct in the harm to health it causes, and is produced by an industry which requires a special form of regulation. Such recognition has been crucial to advances in tobacco control and is known as tobacco exceptionalism.^1^ Paradoxically, this idea may now limit progress in global health by obscuring how hard won lessons may apply to other commercial sectors that damage people’s health.^1^

Tobacco is produced by a highly concentrated and globalized industry, whose behavior over many decades has been shown to have transgressed the most basic ethical responsibilities towards human life in pursuit of profit. The tobacco companies are now accepted as requiring a distinct model of governance, which includes the protection of public health policies from interference because of ‘a fundamental and irreconcilable conflict between the tobacco industry’s interests and public health policy’.^2^ This position is enshrined in a binding United Nations treaty in the World Health Organization Framework Convention on Tobacco Control (WHO FCTC). Tobacco companies’ internal documents constitute an unusually strong form of evidence on their internal machinations. These show that the tobacco companies continue to understand very well that their business interests are compromised by public health policies, and for this reason continue to oppose them strongly.

Alcohol has been used for millennia as an intoxicating drug. Alcohol is different from tobacco in many respects. We do not expect that alcohol will kill half its users, and a minority of drinkers are addicted to alcohol. Instead, the public health burden arises predominantly from the larger number who drink in risky rather than in obviously problematic ways. Alcohol kills fewer people than tobacco globally, approximately 5% compared to 8% of all deaths, though it may be more likely to be under-reported. Alcohol is a component cause of more than 200 diseases, injuries and other health problems, of which more than 40 are wholly attributable. Alcohol is carcinogenic and toxic and kills at younger ages and in some different ways to tobacco, including through violence associated with intoxication.

Alcohol is responsible for social problems, including the need to fund healthcare services. In England the costs to the NHS and wider society are approximately £2.5 and £11 billion respectively for tobacco, compared to £3.5 and £21 billion for alcohol. Both alcohol and tobacco do more damage in socioeconomically deprived populations and exacerbate health inequalities. Public health policies threaten the business interests of alcohol companies, similarly to tobacco companies.

Alcohol is more profitable than other consumer goods apart from tobacco, and profitability in the largest companies approaches that found in tobacco companies. Both industries derive their profitability from unhealthy levels of consumption, and the alcohol industry, particularly in beer and spirits, increasingly resembles the tobacco industry as fewer companies dominate global production.

The tobacco company internal documents reveal how the two industries have long collaborated in advancing shared interests in influencing policy, and importantly, they show that there is a long history of cross ownership that makes it unwise to think of them as separate entities.^3^ More recently, tobacco company parent company Altria strongly supported the third largest merger in corporate history to create the world’s largest alcohol producer, and purchased additional shares to become the second largest shareholder upon completion.^4^

Pricing and availability restrictions are the key policy measures that evidence indicates are most likely to be effective for alcohol, similar to tobacco. Whilst restrictions are widely implemented as public health measures for tobacco, they are much less prominent in national alcohol policies. National alcohol policies frequently emphasize partnerships with industry that would be unthinkable for tobacco companies, yet permit alcohol industry actors wide-ranging influence.

A minority of the world’s population uses either drug, so there are markets to be developed, especially in Low- and Middle-Income Countries. The most recent report on alcohol from WHO reveals no progress in reducing total global per capita alcohol consumption, which is instead forecast to rise.^5^ This will have predictable consequences for death, disease and social problems. It is highly likely that the key Sustainable Development Goals’ target for the harmful use of alcohol will not be met, unless effective policies to protect public health are more widely adopted and implemented.

The achievements of tobacco control rest in part on tobacco exceptionalism. This comparison with alcohol suggests that tobacco may not actually be so different in the challenges posed and the responses required. There have been encouraging signs of integrative perspectives on the global health implications of commercial actors’ business activities and how we address them. The potential value of policy coherence, not only in respect of global health more broadly, but for tobacco control itself has also previously been recognized.^1^ The progress already made in tobacco control means that important lessons are available to protect and improve health more widely.

## Funding

This work was supported by a Wellcome Trust Investigator Award in Humanities and Social Science (200321/Z/15/Z) to JM.

*Conflicts of interest*: None declared.
